# Computational Tumor Infiltration Phenotypes Enable the Spatial and Genomic Analysis of Immune Infiltration in Colorectal Cancer

**DOI:** 10.3389/fonc.2021.552331

**Published:** 2021-03-15

**Authors:** Henrik Failmezger, Natalie Zwing, Achim Tresch, Konstanty Korski, Fabian Schmich

**Affiliations:** ^1^Data Science, Pharma Research and Early Development, Roche Innovation Center Munich, Penzberg, Germany; ^2^Early Biomarker Development Oncology, Pharma Research and Early Development, Roche Innovation Center Munich, Penzberg, Germany; ^3^Faculty of Medicine and University Hospital, University of Cologne, Cologne, Germany; ^4^Excellence Cluster on Cellular Stress Responses in Aging-Associated Diseases (CECAD), University of Cologne, Cologne, Germany; ^5^Center for Data and Simulation Science, University of Cologne, Cologne, Germany

**Keywords:** immunotherapy, machine learning, colorectal cancer, spatial statistics, immune response

## Abstract

Cancer immunotherapy has led to significant therapeutic progress in the treatment of metastatic and formerly untreatable tumors. However, drug response rates are variable and often only a subgroup of patients will show durable response to a treatment. Biomarkers that help to select those patients that will benefit the most from immunotherapy are thus of crucial importance. Here, we aim to identify such biomarkers by investigating the tumor microenvironment, i.e., the interplay between different cell types like immune cells, stromal cells and malignant cells within the tumor and developed a computational method that determines spatial tumor infiltration phenotypes. Our method is based on spatial point pattern analysis of immunohistochemically stained colorectal cancer tumor tissue and accounts for the intra-tumor heterogeneity of immune infiltration. We show that, compared to base-line models, tumor infiltration phenotypes provide significant additional support for the prediction of established biomarkers in a colorectal cancer patient cohort (*n* = 80). Integration of tumor infiltration phenotypes with genetic and genomic data from the same patients furthermore revealed significant associations between spatial infiltration patterns and common mutations in colorectal cancer and gene expression signatures. Based on these associations, we computed novel gene signatures that allow one to predict spatial tumor infiltration patterns from gene expression data only and validated this approach in a separate dataset from the Cancer Genome Atlas.

## Introduction

Cancer immunotherapy is the most promising therapy for many metastatic and formerly untreatable tumors. However, often only a subgroup of patients will benefit from its application. Biomarkers are important predictors of patients' response to a treatment and, moreover, offer new insights into drug mechanisms of action (1, 2). There are different types of biomarkers: For instance, over-expression of genes like PDL1, mutations in specific genes like KRAS or BRAF or impaired DNA mismatch repair (microsatellite instability, MSI) have all been shown to be informative biomarkers in the context of immunotherapy for colorectal cancer (CRC) patients (2). While recently, tumor mutation burden (TMB), i.e., the number of mutations per megabase has been established as another genetic biomarker (3, 4), the field is currently shifting its focus toward the tumor microenvironment (TME) (5–8). The TME is characterized by the complex interaction between malignant tumor cells, immune cells (e.g., CD8+ or CD4+ T cells), as well as stromal cells. It has been shown that the presence of immune cells in malignant tumors is predictive of the success of certain immunotherapies (5, 9). Abundances of immune cells are either determined by gene expression signatures from bulk RNA sequencing (10), or based on immunohistochemical (IHC) staining of tissue samples (11) and subsequent calculation of the immune cell density, i.e., the number of immune cells divided by the tissue area. Traditionally, immune cell densities have been hand-annotated by a pathologist, whereas today, many such workflows have been automated and digitalized using methodology from the fields of Artificial Intelligence and Computer Vision (1, 6, 12).

However, neither gene expression based immune cell abundances, nor immune cell densities derived from IHC provide insight into whether T cells are effectively infiltrating a tumor or whether they are blocked outside of the tumor and concentrated within the stromal tissue. Recently, three major patterns of tumor immune infiltration have been described: *deserted* (no to low T cell abundance), *excluded* (T cells and tumor cells occupy disjoint spatial areas) and *inflamed* (T cells and tumor cells spatially co-localize) (13). It is hypothesized that the spatial arrangement of T cells and tumor cells and in particular the proximity of cytotoxic CD8+ T cells to malignant tumor cells is strongly affecting the immune response (14, 15).

**Figure 1:**
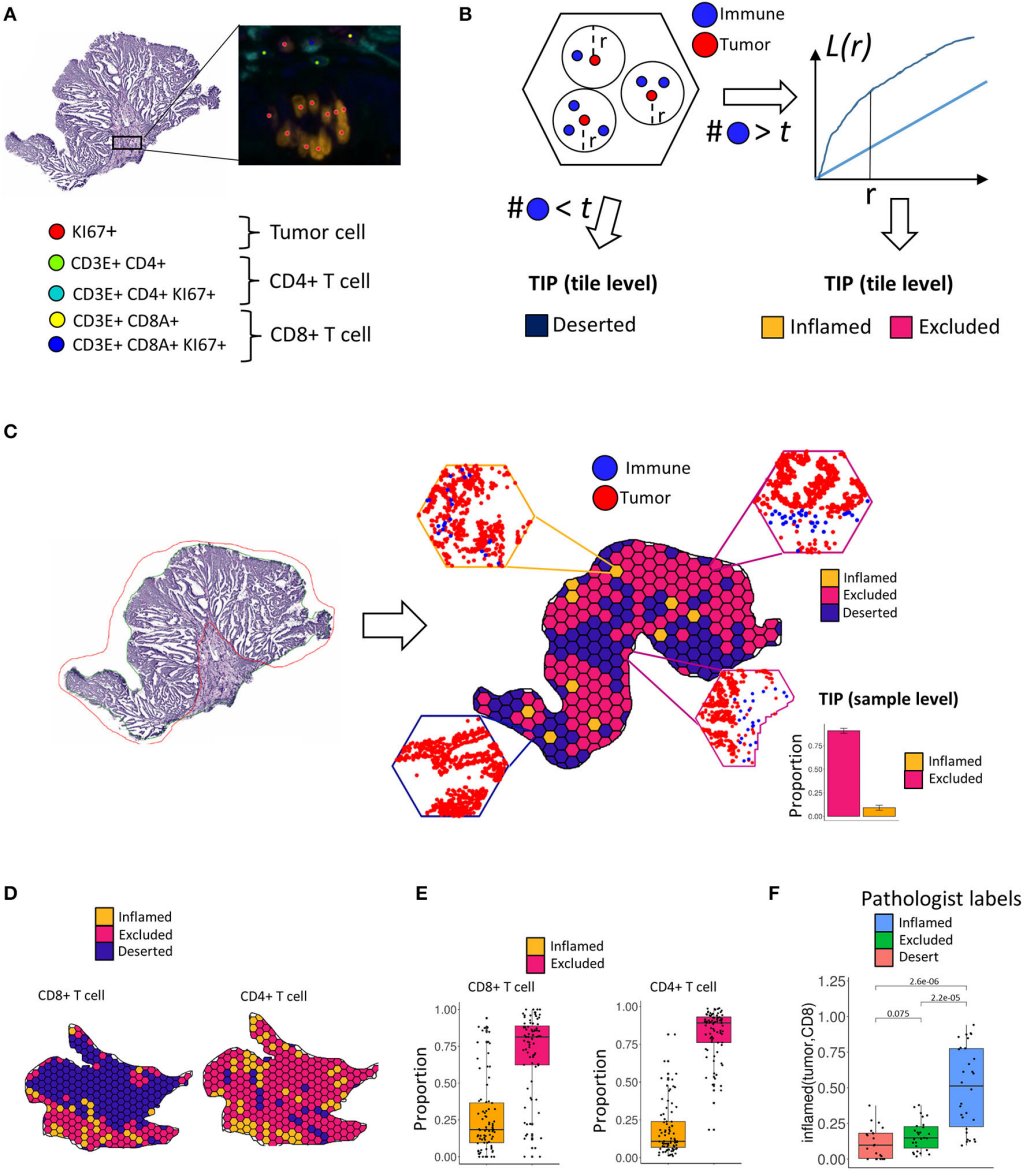
Tumor infiltration phenotypes (TIPs). **(A)** Tumor cells, CD8+ T cells and CD4+ T cells are detected in the immunohistochemistry multiplex stains. **(B)** Assignment of tumor infiltration phenotypes on tile level. If a tile contains less immune cells than a threshold *t*, a tile is classified as deserted. Otherwise, a tile is classified to either an inflamed or excluded tumor infiltration phenotype using the distribution of Ripley's L function as the input to a fused lasso logistic regression model. **(C)** For each tile within a sample, local infiltration patterns are predicted using the trained logistic regression model. The relative frequencies of the classified tiles serve as the measurement for the TIPs of the whole sample. **(D)** On the tile level, TIPs can exhibit high degrees of heterogeneity with respect to different immune infiltration phenotypes. TIPs for CD8 and CD4 immune cells, respectively, computed within the same sample did not always follow a similar distribution. **(E)** On the sample level, both CD8+ T cells as well as CD4+ T cells show higher proportions of exclusion in the cohort. **(F)** The *inflamed (tumor, CD8)* TIP is in strong concordance with manually assigned labels from an expert pathologist.

While *deserted* tumors can be fully characterized based on T cell abundance, the distinction between *excluded* and *inflamed* tumors requires spatial pattern analysis. In this work, we present a novel computational method based on Ripley's L function (16) that, for the first time, is capable to successfully distinguish *excluded* and *inflamed* tumor infiltration phenotypes (TIPs) in CRC tissue (Figure 1B). Ripley's L function has previously been used to characterize the patterning of stromal cells in breast cancer (6) and the patterning of CD8+ T cells and tumor cells in pancreatic cancer (14).

We observed that the patterning of T cells in many tumors showed substantial local heterogeneity across the tissue sample. In order to account for this spatial variation of infiltration phenotypes, we tessellated the sample area into hexagonal tiles (edge length = 375 μm) and calculated the co-localization of T cells and tumor cells per tile. Each tile's specific shape of the L function then served as the input features to a logistic fused lasso regression model (17, 18), that was subsequently trained to classify each tile to either infiltration phenotype (*inflamed* vs. *excluded*). In order to aggregate tile-level information to a quantitative biomarker on the sample level, we computed the respective proportion of tiles for each TIP across the tissue sample. In total, we characterized TIPs for 80 samples from CRC patients. We show that TIPs add valuable information to the prediction of established biomarkers like TMB and MSI and immune cell subpopulations, as defined by gene expression signatures. Further, we developed a novel gene expression deconvolution scheme, accounting for differently sized populations of immune- and tumor cells within a sample, which allowed us to infer distinct gene expression signatures that predict the spatial tumor infiltration phenotypes of a sample. We validated the prediction of these phenotypes from gene signatures in the Cancer Genome Atlas.

## Materials and Methods

### Histological Data

Surgical specimens from primary colorectal tumors were procured. They were collected from consented patients (informed consent) and under approval from the respective Institutional Review Board, National Ethics Committee, or equivalent agency. The samples had been fixed in formalin, embedded in paraffin and archived prior to shipment.

A total amount of 80 tissue specimens was obtained. The cells in the sections were stained by fluorescent immunohistochemistry with the markers Ki67+ (30-9, Ventana Medical Systems), CD3+/CD4+ (2GV6, Ventana Medical Systems) and CD3+/CD8+ (SP239, Spring Biosciences) (19). The slides were digitized with a Zeiss Axio Scan.Z1 at 20x magnification, resulting in a pixel resolution of 325 nm.

In order to determine the MSI status, the slides of the tissue samples were stained and assessed for the presence or absence of four mismatch repair (MMR) proteins, MLH1 (M1, Ventana), MSH2 (G219-1129, Ventana), MSH6 (SP93, Ventana), and PMS2 (A16-4, Ventana). Tumors with loss of one or more of the MMR proteins are considered MSI, whereas intact MMR staining is classified as MSS (20). 24% of the patients were MSI (Table 1) and most patients were at a late tumor stage (stage 3: 20%, stage 4: 64%) (Table 1). Tumor mutational burden was calculated as the number of mutations per megabase.

**Table 1:** Characteristics of the dataset.

**Characteristic**	***N* = 80**
**Age**^**a**^	69 (60,77)
**Tumor grade**	
G1	9 (11%)
G2	45 (56%)
G3	26 (32%)
**Gender**	
Female	40 (50%)
Male	40 (50%)
**MMR status**	
MSI	19 (24%)
MSS	61 (76%)
**Tumor stage**	
I	2 (2.5%)
II	11 (14%)
III	16 (20%)
IV	51 (64%)

a *Statistics presented: median (IQR)*.

### Image Processing and Analysis

Tumor and immune cells were detected and classified by a proprietary machine learning algorithm based on color, intensity, texture, object shape. For each cell, we obtained its position and its cell type (Figure 1A): Tumor cells (stained by KI67+), CD4 cells (stained by CD3+CD4+ or CD3+CD4+Ki67+), CD8 cells (stained by CD3+CD8A+ or CD3+CD8A+KI67+). An expert pathologist, who also verified the results of the algorithm, manually annotated tumor regions. Only cells within the manually labeled tumor region were used for analysis. Furthermore, we excluded areas within each tile (hexagons, side length = 50 μm) with less than two cells in order to avoid artifacts caused by these regions, e.g., holes could bias the computation of the Ripley's L function (Supplementary Figure 1A).

### Genomic Data

The mutation dataset consisted of 373 cancer and immune related genes based on the gene panel of FoundationOne (21). Mutation data was available for 75 patients. For the mutation analysis, we kept only genes that had a mutation in at least 20% of the patients, resulting in a dataset of 19 genes (Supplementary Table 6).

For generating the RNAseq data for the tissue samples (22), genomic DNA and total RNA were purified from 10 μm thick FFPET curls using the AllPrep DNA/RNA FFPE Kit (Qiagen Cat No./ID: 80234). The Qubit instrument was used to assess the RNA samples for quality and quantity and the Agilent Bioanalyzer was used to determine the degradation of the RNA samples (DV200 value). To further generate the sequencing library, the hybridization-based Illumina TruSeq RNA Access method was performed, with first preparation of the total RNA library and second library enrichment for coding RNA. Finally, normalized libraries were sequenced using the Illumina sequencing-by-synthesis platform, with a sequencing protocol of 50 bp paired-end sequencing and total read depth of 25M reads per sample (22). The data has been deposited as part of a previous publication in the Gene Expression Omnibus (https://www.ncbi.nlm.nih.gov/geo/query/acc.cgi?acc=GSE152395). For further analyses, we selected from the gene expression data all genes that had no missing value in any patient resulting in RNAseq count data for 14,634 genes and 66 patients. Signatures for immune subpopulations were derived from Angelova et al. (23), while the effect size of association of a signature and a patient sample was calculated by gene set enrichment analysis using the gCMAP package (24, 25).

Gene expression data for the TCGA-COAD and TCGA-READ datasets from the Cancer Genome Atlas (TCGA) were retrieved by TCGAbiolinks (26). Tumors with the classification: *Splenic flexure of colon, Sigmoid colon, Descending colon, Rectosigmoid junction* were considered as left side tumors. Tumors with the classification: *Ascending colon, Transverse colon, Cecum, Hepatic flexure of colon* were considered as right side tumors.

### Pathway Mutation Score

Pathways were retrieved from the KEGG database (27). We selected pathways for which the overlap between the pathway genes and our mutation gene set (373 genes) was at least 40% which resulted in 14 pathways (**Figure 4B**).

The pathway mutation score *m*_*i,p*_ for a patient *i* and pathway p was calculated as:

$$\begin{array}{l} m_{i,p}=\frac{\#mutated\;genes\;for\;patient\;i\;in\;pathway\;p}{\#genes\;in\;pathway\;p} \end{array}$$

### Spatial Statistics Measuring Interaction of Cells

Tumor tissue was tessellated into hexagonal tiles with a side length of 375 μm. Based on spatial x and y coordinates of each cell, as derived from image processing of the imaged tissue, Ripley's L function was calculated for different combinations of cell types *i* and *j* within each tile. *L*_*ij*_(*r*) is defined as: $L_{ij}\left(r\right)=\sqrt{\frac{K_{ij}\left(r\right)}{\pi }}$, where

$K_{ij}\left(r\right)=\frac{1}{\lambda_{j}}\;*\;E\left[t\left(u,r,X^{\left(j\right)}\right)|u\in X^{\left(i\right)}\right]$ (28), λ_*j*_ is the density of cell type *j* and

$t\left(u,r,X^{\left(j\right)}\right)=\overset{n\left(x^{\left(j\right)}\right)}{\underset{k=1}{\sum }}1\left\{0<\;\|u-x_{k}\;\|\leq r\right\}$ is the number of cells of type *j* that lie within a distance *r* of the location *u* (28). In what follows, *i* always denotes tumor cells, and *j* either denotes CD8 or CD4 cells, i.e., we quantify the proximity of immune cells to tumor cells from the perspective of each tumor cell. The expectation value in Ripley's L function is estimated by empirical cell frequencies. Since the area under scrutiny however is finite, it is necessary to apply isotropic edge correction (28) to obtain an unbiased estimate.

### Calculation of Tumor Infiltration Phenotypes

In order to cast Ripley's L curves into the phenotypes *inflamed* vs. *excluded*, we trained a 1d logistic fused lasso regression model based on each tile's feature vector. Let *S* be the number of samples (tiles). Let *y*_*s*_ ∈ (*inflamed, excluded*), s = 1,…,S be the ground truth labels of the tiles. A feature vector *x*_*sr*_ = *L*_*sij*_(*r*) − *r*, with *r* ranging over an equidistant grid of n values in the range of 1 to 338 μm, is calculated for every tile. Here *L*_*sij*_(*r*) is the observed L function in the tile for cell types *i* and *j* and *r* is the expected value of the L function under the assumption that there is independence between the two point patterns. The logistic fused lasso regression model is defined as:

$$\begin{array}{l} \hat{\beta }={\mathrm{argmin}}_{\beta \in {\mathbb{R}}^{n}}-\frac{1}{S}\overset{S}{\underset{s=1}{\sum }}\{y_{s}\left(\beta_{0}+\beta^{T}x_{s}\right)-\log\left(1+e^{\beta_{0}+\beta^{T}x_{s}}\right)\}+\\ \lambda_{1}\overset{n-1}{\underset{r=1}{\sum }}|\beta_{r+1}-\beta_{r}|+\lambda_{2}\overset{n}{\underset{r=1}{\sum }}|\beta_{r}| \end{array}$$

(17, 18)

The ground truth labels *y*_*s*_, required to fit the logistic regression model, were annotated by an expert pathologist. The penalty term λ_1_ in the model enforces smoothness of neighboring coefficients, i.e., points on the Ripley's L curve, and makes it particularly suitable for a situation, where we expect high degrees of correlation between consecutive points in this 1d-sequential feature space. The fused lasso regression model was fit on a balanced training set of *n* = 118 tiles. As fluorescent immunohistochemistry multiplex stainings are difficult to analyze visually this ground truth data was created based on a KI67/CD8 duplex staining. We chose a side length of 375 μm for the tiles, which ensured a sufficient number of tiles per tissue, but was also large enough for the pathologist to evaluate the patterning of the cells in the tile. A minimum of more than five immune cells was required for a pathologist to reliably distinguish the infiltration patterns *inflamed* vs. *excluded*. Therefore, if the number of immune cells within a tile was smaller than or equal to five, we set its infiltration pattern to *deserted*.

Estimates of Ripley's L function become unstable at radii close to the maximum tile size (375 μm). We therefore restricted our feature vector to values of r from 1 to 338 μm, the latter being about 90% of the tile size. The parameters λ_1_ and λ_2_ that regularize both the difference of consecutive coefficients as well the coefficients by itself were determined by five-fold cross validation (Supplementary Figure 1B). We use the notation *inflamed (i,j)* and *excluded (i,j)* to describe the TIPs, both on the tile level and on the whole tissue level. On the tile level the TIP *inflamed (tumor, CD8)*, for instance, represents the classification result of the fused lasso regression model, whereas on the whole tissue level it represents the fraction of tiles classified *inflamed*. In order to make the calculations robust against the exact position of the tiles, we shifted the tile grid by 10% in all directions that are integer multiples of 90° and repeated the calculation of TIPs. The resulting four proportions and the original one are averaged to obtain the final TIP.

### Multivariate Regression Analysis for Biomarker Validation

We evaluated the predictive capacity of TIPs for established biomarkers and the gene expression signatures against a base model consisting of the covariates *Age, Tumor Stage* > *IV, CD8*+ *T cell density and CD4*+ *T cell density*. We selected *inflamed (tumor, CD8)* and *inflamed (tumor, CD4)* for evaluation.

In the process of building the extended model containing the TIPs, we first applied a univariate regression against an established CRC biomarker. We then checked if any of the tumor infiltration phenotypes had a Pearson correlation coefficient larger than 0.6 with another TIP. In this case, in order to avoid correlated co-variates, we selected the TIPs that performed better in the univariate regression. We added all uncorrelated TIPs to the base model, reduced the model by stepwise regression analysis (forward-backward selection) and tested if the advanced model significantly improved over the base model (likelihood ratio (LR) test).

### Gene Expression Deconvolution

Bulk RNAseq gene expression data is derived from a mixture of different cell types, most prominently immune cells and cancer cells in our application. Therefore, we implemented a gene expression deconvolution scheme in order to derive gene expression values with reduced bias from the cellular composition of the bulked measurement, i.e., highly imbalanced abundances of different cell types.

Let G be a set of genes and S a set of samples, let

$$\begin{array}{l} X=\left(x_{g,s}\right)\in {\mathbb{R}}^{GxS} \end{array}$$

be the expression matrix normalized first by samples and afterwards by genes. The technical noise of gene expression data is following a poison distribution (29) in which the variance equals the mean, thus we divided every gene by the square root of its mean (i.e., variance). By variance stabilizing each gene, we ensured that the linear approximation of all components is not dominated by a few highly expressed genes and that the following convex optimization has a physical interpretation as a mixture of cells.

Let λ = (λ_*s*_; *s* ∈ *S*), 0 ≤ λ ≤ 1 be the vector of relative abundances of immune cells.

The task is to infer a prototype cancer cell expression profile *C* = (*c*_*g*_; *g* ∈ *G*) and an immune expression profile *I* = (*i*_*g*_; *g* ∈ *G*) such that X is approximated by a convex combination $\tilde{\text{X}}$ of I and C:

$$\begin{array}{l} X\approx \tilde{X}=I\;*\;\lambda^{T}+C\;*\;{\left(1-\lambda \right)}^{T} \end{array}$$

This can be solved using constrained convex optimization:

$$\begin{array}{l} \underset{I,C}{\text{argmin}}{\left(X-\tilde{X}\left(I,C\right)\right)}^{2} \end{array}$$

Subject to the constraints:

$$\begin{array}{l} i_{g}\geq 0;g\in G,\;\underset{g}{\sum }i_{g}=1\\ c_{g}\geq 0;g\in G,\;\underset{g}{\sum }c_{g}=1 \end{array}$$

### Gene Signatures

For every sample, *s* ∈ *S* the estimated value ${\tilde{x}}_{g,s},\;s\in S\;$reveals the closest approximation of the value *x*_*g,s*_, *s* ∈ *S* using only a mixture of the global cancer profile C and the global immune profile I. Genes that influence the spatial localization of immune cells (= the Tumor Infiltration Phenotype) should be independent from the ${\tilde{x}}_{g,s},\;s\in S\;$pattern. A measurement from a TIP *a* = (*a*_*s*_; *s* ∈ *S*) is thus associated to $r_{g,s}=x_{g,s}-{\tilde{x}}_{g,s},\;r\epsilon R.\;$This association of a TIP *a* to the expression of a certain gene *g* can be estimated by linear regression:

$$\begin{array}{l} a_{s}=\beta_{g}\;*\;r_{g,s},\;r\epsilon R \end{array}$$

Genes that showed a significant association with a TIP (Bonferroni multiple testing correction) became part of this TIP's gene expression signature.

Using the expression values of the genes in the signature as covariates, tumor infiltration phenotypes can be explained by gene expression alone. Therefore, we trained a support vector machine for the prediction of tumor infiltration phenotypes from gene signatures. The gamma and the cost parameter were tuned by a grid search (cost=4, gamma=0.0009765625).

### Other Statistical Methods and Data Availability

All statistical analysis was carried out using R ≤3.5.1 (R Core Team 2013). The R package spatstat was used for spatial analysis (28, 30, 31). The R package penalized was used for the fused lasso model (32). GGplot2 was used for visualization (33). The lmerTest package was used for the likelihood ratio test (34). We used the package lol for the stability selection approach (35).

CVXR was used for solving the gene expression deconvolution (36). TopGO was used for differential gene expression analysis (37). The R-package e1071 was used for the training of the support vector machine (38). Example sourcecode as well as all spatial point patterns can be found in supplementary data (Supplementary File 2–6).

## Results

### Computation of Tumor Infiltration Patterns From IHC Stained Tissue

In order to determine local immune infiltration patterns in the tumor, CD8+ T cells, CD4+ T cells and tumor cells were first detected by an automated image processing pipeline (Methods, Figure 1A). Subsequently, in order to account for the spatial heterogeneity of immune infiltration, we separated the tumor sample into hexagonal tiles (Methods). For each tile, we computed Ripley's L function across pairs of tumor and immune cells within the tile (Methods). Ripley's L function is a cumulative function that determines, from the perspective of one type of cell, the number of neighbors of another type of cell within a certain distance r (Figure 1B, Methods). The shape of the L function is indicative of a segregating or an aggregating relationship between the two cell types.

After computing the L function, we classified each tile within the sample into local infiltration patterns (Figure 1B, Methods) using a fused lasso logistic regression model (Methods). The fused lasso regression model was trained on tiles that were manually labeled by a pathologist and resulted in a cross-validation accuracy of 85% (Supplementary Figure 1B, Methods).

The TIPs *inflamed (tumor, CD8)* and *inflamed (tumor, CD4)* represent patterns where tumor cells and immune cells are co-clustered, whereas the TIPs *excluded (tumor, CD8)* and *excluded (tumor, CD4)* represent patterns where immune cells are segregated from tumor cells.

We found that the tile-level distribution of TIPs varied substantially, indicating high degrees of intra sample heterogeneity. For the same sample, we also identified different spatial infiltration patterns for CD8+ T cells and CD4+ T cells, respectively (Figure 1D). On the sample level we detected *excluded (tumor, CD8)* and *excluded (tumor, CD4)* to be the most frequent TIPs in the cohort (Figure 1E). We compared the TIP *inflamed (tumor, CD8)* to immune infiltration phenotypes manually assigned by an expert pathologist based on inspection of the whole tissue sample. Samples labeled inflamed by the pathologist also showed significantly higher fractions (*p* = 2.2e-05) of the TIP *inflamed (tumor, CD8)*, indicating strong concordance between predicted infiltration patterns and experts' ground truth annotations (Figure 1F).

### Tumor Infiltration Phenotypes Predict Established, Prognostic Biomarkers in Colorectal Cancer

Patient survival and response in colorectal cancer is well-known to be linked with the presence of certain prognostic biomarkers, like the microsatellite instability (MSI) status, tumor mutational burden (TMB) and the expression of immune cell signatures. We investigated the association of TIPs with these biomarkers and evaluated if TIPs could act as their independent predictors.

**Figure 2:**
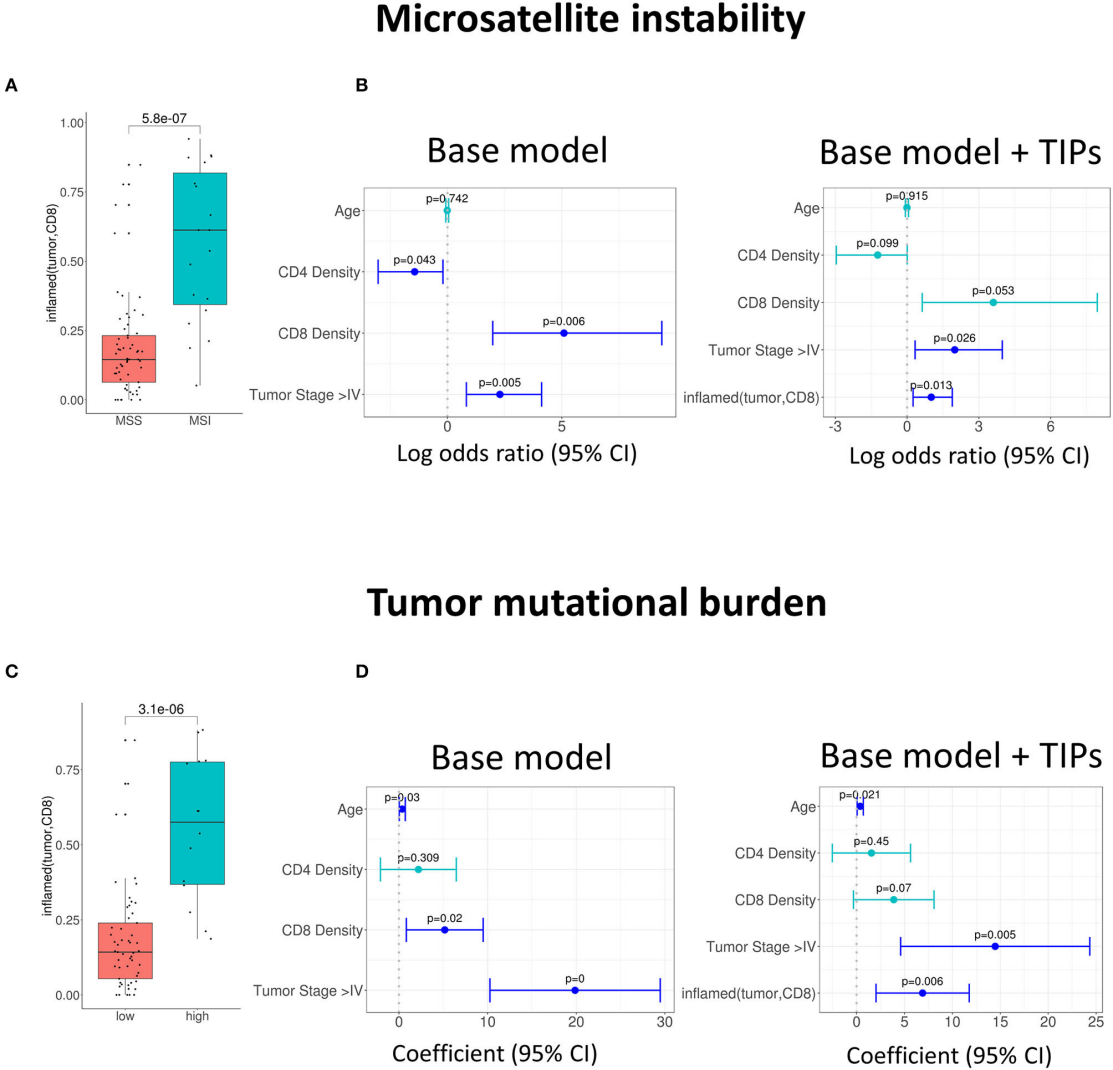
Association of tumor infiltration phenotypes (TIP) with established biomarkers in colorectal cancer. **(A)** Univariate and multivariate **(B)** association of *inflamed (tumor, CD8)* with the MSI status (logistic regression). Multivariate prediction of the MSI status is significantly improved, when adding *inflamed (tumor, CD8*) to the base model [likelihood ratio (LR) test = 0.009]. **(C)** Univariate and multivariate **(D)** association of *inflamed (tumor, CD8)* with tumor mutational burden (TMB). The TMB in **(C)** is grouped into high and low by a threshold of 20. Multivariate prediction of TMB is significantly improved when using the *inflamed (tumor, CD8)* as an additional biomarker [likelihood ratio (LR) test = 0.004]. In all forest plots, the X-axis represents the log odds ratio (MSI) or the regression coefficients (TMB) and 95% intervals (whiskers). The dashed vertical line represents a regression coefficient or and log odds ratio of 0. Significant covariates are indicated in blue, non-significant covariates are indicated in turquoise.

We found a positive univariate association of the TIP *inflamed (tumor, CD8*) with the patients' microsatellite instability (MSI) status (*p* = 5.8e-07, Figure 2A). In order to quantify the observed association, we investigated, if TIPs provide orthogonal, non-redundant information for the prediction of MSI and tumor mutational burden (TMB) when compared to a baseline model consisting of the biomarkers CD8+ T cell density, CD4+ T cell density, Age and Tumor stage (≥ Stage 4). All computationally derived TIPs were added as covariates to the base model. Subsequently, the number of covariates was iteratively reduced using stepwise regression (Methods). For the prediction of MSI, *inflamed (tumor, CD8)* was kept as a covariate in the extended model (Figure 2B, Supplementary Table 1), improving the overall model significantly (*p* = 0.009).

Similarly, the TIP *inflamed (tumor, CD8)* was associated positively with high TMB [*p* = 3.1e-06, a TMB threshold of 20 was used for the assignment to the TMB high or low group (39), Figure 2C]. When compared to the base model, the prediction of TMB was again significantly improved by adding *inflamed (tumor, CD8)* (*p* = 0.004, Figure 2D, Supplementary Table 2).

**Figure 3:**
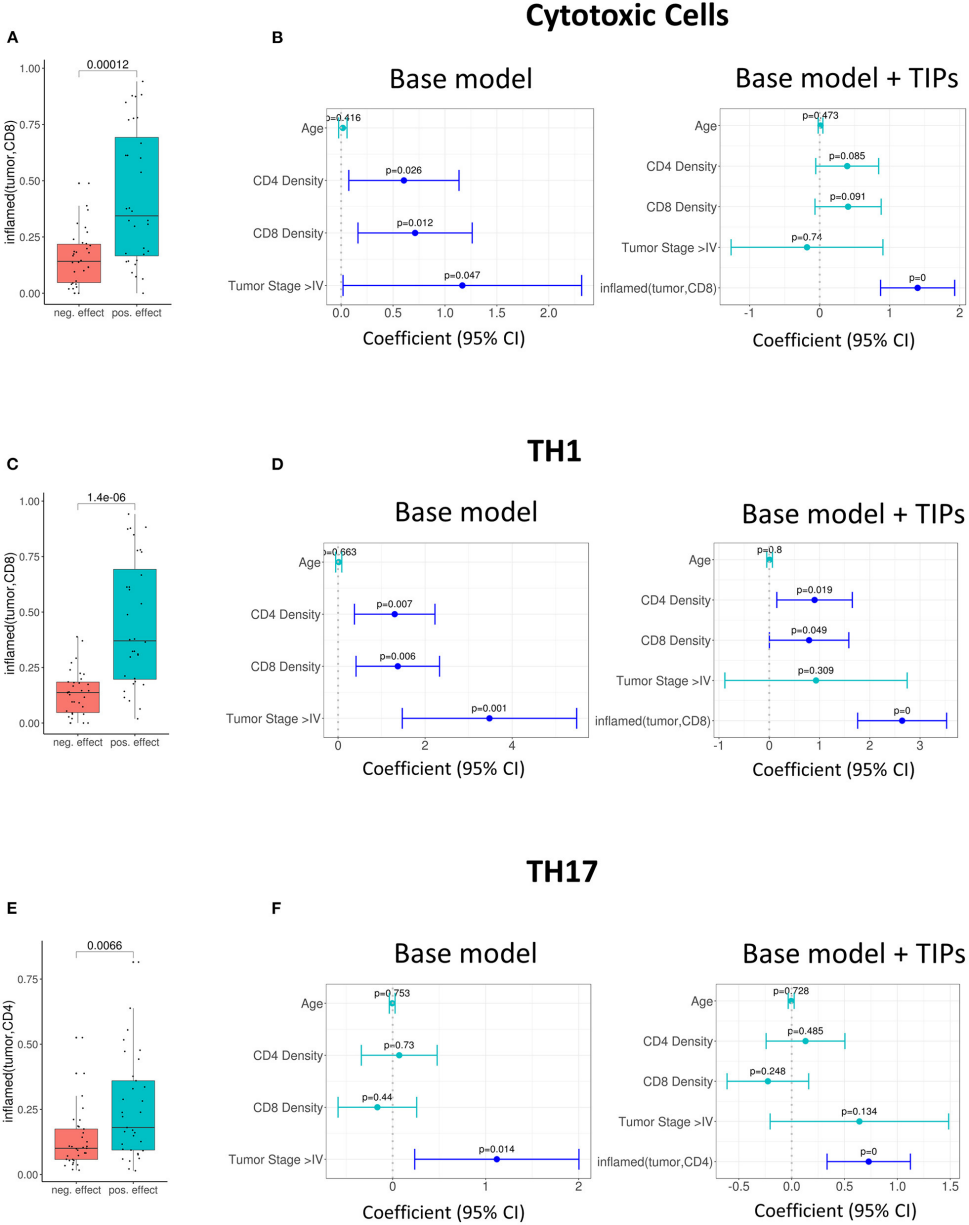
Tumor infiltration phenotypes (TIPs) predict immune cell signature effect sizes. **(A)** Univariate association of *inflamed (tumor, CD8)* with positive and negative effect size of the cytotoxic immune cell signature (Wilcoxon test). **(B)** Multivariate association of *inflamed (tumor, CD8)* with the effect size of cytotoxic immune cell signature. *Inflamed (tumor, CD8)* improves the base model significantly [likelihood ratio (LR) test = 5.01e-0.7]. **(C)** Univariate association of *inflamed (tumor, CD8)* with positive and negative effect size of T-helper Cell 1 immune cell signature. **(D)** Multivariate association of *inflamed (tumor, CD8)* with the effect size of T-helper Cell 1 immune cell signature. The base model is significantly improved [likelihood ratio (LR) test = 2.8e-08]. **(E)** Univariate association of *inflamed (tumor, CD4)* with positive and negative effect size of T helper cell 17 immune cell signature (Wilcoxon test). **(F)** Multivariate association of *inflamed (tumor, CD4)* with the effect size of T-helper Cell 17 immune cell signature [likelihood ratio (LR) test = 0.0002]. In all forest plots, the X-axis represents the regression coefficients and 95% intervals (whiskers). The dashed vertical line represents a regression coefficient of 0. Significant covariates are indicated in blue, non-significant covariates are indicated in turquoise.

Further, we investigated the association of TIPs with subpopulations of tumor infiltrating lymphocytes. We calculated the effect size of three immune signatures [cytotoxic T cell, T helper cell 1 (TH1) and T helper cell 17 (TH17) (23)] from matched gene expression data using gene set enrichment analysis (see Methods). There was an association between *inflamed (tumor, CD8)* and the sign of cytotoxic and TH1 immune signature's effect size (Cytotoxic cells: *p* = 0.00012, Figure 3A, TH1: *p* = 1.4e-06, Figure 3C) and between *inflamed (tumor, CD4)* and the effect size of the TH17 signature (*p* = 0.007, Figure 3E).

In order to quantify if the TIPs provide additional, non-redundant information for the prediction of the effect sizes of the three immune signatures, we again added them to the base model and reduced the number of covariates by stepwise regression (Methods).

For the prediction of the immune signature of cytotoxic cells, *inflamed (tumor, CD8*) was kept in the model. The addition of the TIP improved the model significantly (*p* = 5.01e-07, Figure 3B, Supplementary Table 3).

For the prediction of the immune signature of TH1 cells, again *inflamed (tumor, CD8*) was kept in the model (Figure 3D, Supplementary Table 4). For the prediction of the immune signature of TH17 cells, *inflamed (tumor, CD4)* was kept in the model (Figure 3F, Supplementary Table 5). Both models were significantly improved by adding the TIPs (TH1 cells: *p* = 2.8e-08, TH17 cells: *p* = 0.0002).

### Tumor Infiltration Phenotypes Are Associated With Mutations on the Single-Gene and Pathway Level

**Figure 4:**
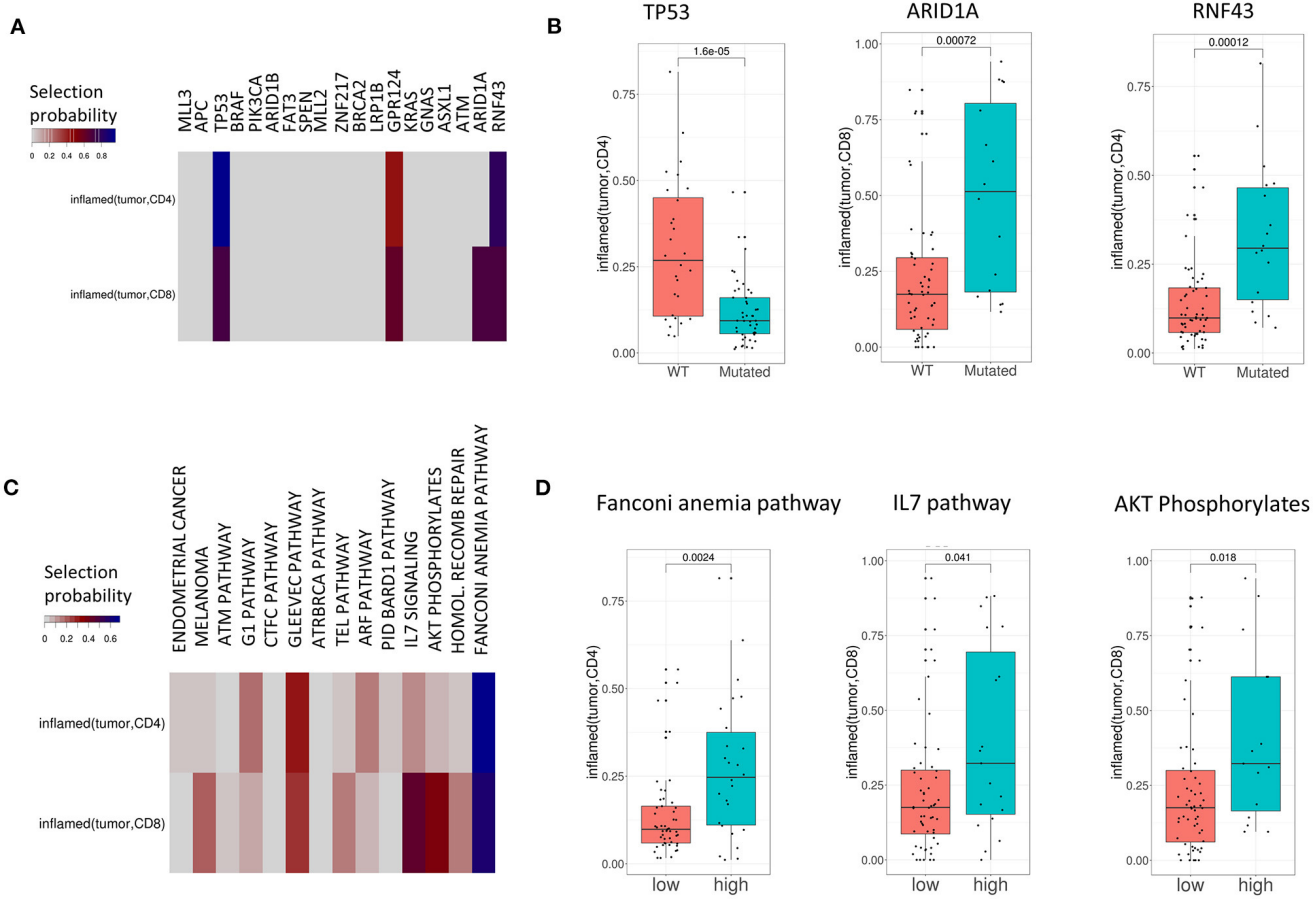
TIPs are associated with mutations both on the single-gene and pathway level. **(A)** Associations between mutations and TIPs by Lasso stability selection. The coefficients represent the selection probability in 200 runs of the Lasso model. TP53, ARID1A, and RNF43 showed the strongest associations with a TIP. **(B)** Associations of mutated genes with TIPS (Wilcoxon test). **(C)** Associations between mutations in signaling pathways (pathway mutation scores) and TIPs by Lasso stability selection. The coefficients represent the selection probability in 200 runs of the Lasso model. The Fanconi Anemia pathway, the IL7 signaling pathway and the AKT phosphorylates pathway showed the strongest associations with TIPs. **(D)** Association of high and low pathway mutation scores with TIPs (Wilcoxon test). Pathway mutation scores are split into low and high at the median.

We investigated the association of common mutations in CRC with TIPs. For this purpose, we implemented a Lasso regularized regression model that explains each TIP (*inflamed* and *excluded*) using the patients' mutation profiles as covariates (Figure 4). The analysis was performed both on the single-gene level (Figure 4A) and the pathway level (Figure 4C) using single-gene mutations that occurred in at least 20% of patient samples and 14 selected pathways with at least 40% overlap with the set of mutated genes, respectively (see Methods and Supplementary Table 6). In order to robustly select pathways and mutations that are associated with TIPs, we used a stability selection approach for the Lasso model (35, 40), using the selection probability as an indicator of the association strength of a gene or a pathway with a TIP.

On the single-gene level, four out of 19 genes were selected in at least 40% of the runs (TP53, GPR124, ARID1A, and RNF43) reflecting the importance of these genes in explaining different TIPs. The tumor suppressor gene p53 (*p* = 1.6e-05, Wilcoxon Test, Figure 4B) and the kinases ARID1A (*p* = 0.0007, Wilcoxon Test, Figure 4B) and RNF43 (*p* = 0.00012, Wilcoxon Test, Figure 4B) showed the strongest single-gene associations with a TIP in terms of selection probability (Figure 4A), indicating these genes to be relevant factors for the TIPs *inflamed (tumor, CD4*) (TP53, RNF43) and *inflamed (tumor, CD8)* (ARID1A), respectively. On the pathway level, 12 of the 14 pathways were found to be associated with at least one TIP. Here, mutations in the *Fanconi Anemia* pathway (*p* = 0.0024, Wilcoxon Test, Figure 4D), the *IL-7 signaling* pathway (*p* = 0.041, Wilcoxon Test, Figure 4D) and the *AKT* pathway (*p* = 0.018, Wilcoxon Test, Figure 4D) showed the strongest associations with a TIP [*inflamed (tumor, CD4*): *Fanconi Anemia* pathway, *inflamed (tumor, CD8*): *IL-7 signaling* pathway, *AKT* pathway].

### Tumor Infiltration Phenotypes Are Associated With Gene Expression Changes in Immune-Related Pathways

**Figure 5:**
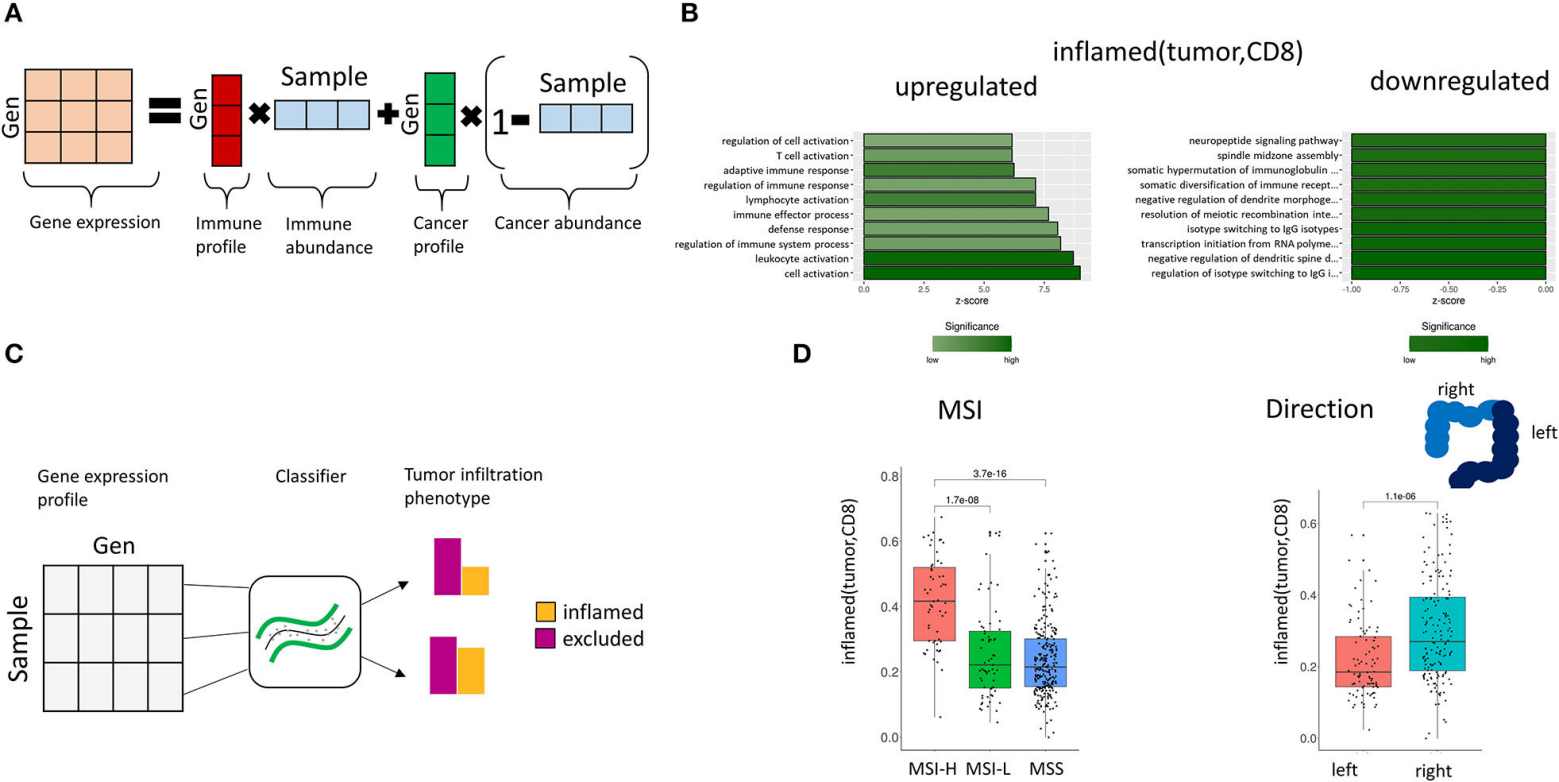
Association of gene expression and TIPs. **(A)** Deconvolution of bulk gene expression data using cell proportions from image processing. The observed gene expression data is approximated by the mixture composition of immune and cancer cells. **(B)** Enriched GO terms in the gene expression profile for *inflamed (tumor, CD8)*. The z-score indicates the gene count for the specific term. **(C)** Prediction of TIPs from gene expression data. A support vector machine classifier is trained with the sample's genes expression values as features and the TIP value as response, which allows the prediction of spatial immune infiltration phenotypes from genomic data. **(D)** Prediction of TIPs from gene expression data in a separate cohort of CRC patients (TCGA) and association to microsatellite instability (MSI) and tumor laterality, respectively.

We hypothesized that TIPs evoke distinct gene regulatory programs in lymphocytes and tumor cells, respectively. Therefore, we were interested in detecting differences in gene expression that cannot be explained by variation in cell type composition, but which result from changes in pathway activity in either lymphocytes or tumor cells. For this purpose, we developed an approach for the deconvolution of gene expression, separating variation in gene expression due to cell proportions from changes in gene expression resulting from distinct regulatory programs (Figure 5A, Methods). Afterwards, we determined the association of TIPs to differentially expressed genes by linear regression analysis, with the TIP as the response and the gene expression values as covariates. Genes with regression coefficients significantly different from zero (*p* < 0.05 after Bonferroni multiple testing correction) were added to the respective TIP's gene signature.

We found 303 genes to be significantly associated with *inflamed (tumor, CD8)* (Figure 5B, Supplementary Table 7), and 87 genes to be associated with *inflamed (tumor, CD4*) (Supplementary Figure 2, Supplementary Table 8).

Gene ontology (GO) term enrichment analysis on the upregulated genes for the TIP *inflamed (tumor, CD8)* revealed terms associated with the immune system (including immune response, leukocyte activation, immune effector process) (Figure 5B). The downregulated genes were found to be associated to dendrite morphogenesis regulation and transcription initiation. Among the significantly downregulated genes we identified the DNA mismatch repair protein MLH1 (41), the genes CRCP and ZXDA with roles in DNA transcription regulation and BBS10 with a role in protein folding (42, 43). The most significant upregulated genes included histocompatibility antigens like HLA-DMA, HLA-DPA1, HLA-DRA, HLA-DPB1, HLA-DMB, and genes with a role in immune response (CD74, TCIRG1, RAC2, ITGAE).

GO terms in the gene signature for *inflamed (tumor, CD4)* were enriched for neutrophil migration, neutrophil chemotaxis and regulation of dendritic spine (Supplementary Figure 2).

### Genetic Signatures Predict Tumor Infiltration Phenotypes

Gene signatures allow extending the definition of TIPs from the tissue to the molecular level. In order to test if the derived TIP-specific gene signatures can also be used to predict TIPs in new datasets with missing IHC image data, we obtained two genomic datasets from CRC patients, TCGA-COAD (*n* = 298) and TCGA-READ (*n* = 98), using the Cancer Genome Atlas (TCGA).

First, we trained a support vector machine on the original CRC (*n* = 80) dataset with the TIPs as response variables and the genes of the corresponding gene signature as features (Figure 5C), learning the association between gene expression and TIPs. Afterwards we applied this model to predict TIPs in the genomic datasets from TCGA. We found the predicted TIP *inflamed (tumor, CD8)* to be significantly higher in MSI-High patients than in MSI-Low or MSS (Microsatellite stable) patients as defined in TCGA (Figure 5D, *p* = 3.7e-16). Further, we found the TIP *inflamed (tumor, CD8*) to be significantly higher in right side tumors as compared to left side tumors (Figure 5D, *p* = 1.1e-06), confirming the hypothesis of right side tumors having a stronger immune infiltrated tumor microenvironment (44).

## Discussion

The quantification of spatial tumor immune infiltration has great potential to form patient enrichment strategies for immunotherapy, or to help to better understand the mode of action of a drug in early clinical trials. For instance, the co-localization of immune cells and tumor cells has been proven to be connected to beneficial survival in many tumor types (14, 15). Here, we have developed a method that is based on the three basic immune infiltration patterns inflamed/excluded/deserted but, in contrast to other approaches, is independent of manual annotations and thus allows the automated, quantitative identification from immunohistochemically stained samples. In comparison to other studies that used spatial statistics to infer spatial patterns of immune and tumor cells (14, 45), our method considers the intra-tumor heterogeneity of immune infiltration. This is achieved by dividing the tissue into tiles and applying Ripley's L statistic in order to classify the tiles according to their local immune infiltration pattern. The proportion of the local immune infiltration patterns in the sample serves as our measurement of the global tumor infiltration phenotype (TIP).

We have demonstrated that TIPs are associated with the MSI and TMB status of a patient. While it has been shown previously that MSI and high TMB are associated with a higher immune cell density (46), we could show that they are also associated with an increased co-localization between CD8+ T cells and tumor cells. Moreover, TIPs were shown to be independent from other covariates like cell density, age and tumor stage and thus served as an additional factor for the prediction of the MSI and TMB status, respectively.

Notably, the effect size of the immune signature for Cytotoxic T cells was correlated with increased co-localization between tumor and CD8+ T cells. This highlights the great potential to combine spatial infiltration phenotypes with phenotypes derived from gene expression, like the subpopulation of cytotoxic cells in order to predict the outcome of immunotherapy. Nevertheless, the prognostic and predictive relevance of phenotype combinations must be determined in future studies.

Furthermore, we found TIPs to be associated with common mutations in CRC. It has been shown that immune infiltration is triggered by somatic mutations. Whereas, on the one hand mutations can induce immune escape mechanisms in the tumor cells (47), they can on the other hand also form neoantigens that are detected by the immune system (48). We showed that mutations in p53 resulted in reduced co-localization between tumor and CD8+ T cells whereas mutations in ARID1A and RNF43 had the opposite effect. A reason may be that p53 dysfunction leads to immunosuppression and immune evasion (49) whereas mutations in ARID1A and RNF43 might act as neoantigens. Notably, mutations in the Fanconi Anemia pathway were associated with an increased co-localization between tumor cells and CD4 cells. As the Fanconi pathway is a DNA mismatch repair pathway (50), perturbation of this pathway causes an accumulation of replication errors, making cancer cells more easily recognizable by cytotoxic cells (51). Moreover, mutations in the AKT pathway and the IL7 signaling pathway were connected with TIPs. The AKT pathway is a cell signaling pathway. It is well-know that activation of AKT is a mechanism of tumor immune evasion (52), which is corresponding to the observation that high mutation rates in this pathway were in our analysis connected with more co-localization between tumor cells and CD8 cells. Interleukin-7 is an important cytokine that stimulates B and T cell development. However, it has been shown that in tumor cells IL-7 is associated with increased aggressiveness of solid tumors and enlarged metastasis rate (53, 54). Although the association of high mutation rates in these pathways with TIPs is less obvious, it shows the potential of TIPs for the characterization of the immune response when combined with genomic data.

Following this line of thought, we integrated spatial immune infiltration patterns, as represented by the TIPs, with gene expression data. Using a gene expression deconvolution scheme in combination with a regression model, we were able to find specific gene signatures for TIPs. We found upregulated genes in the signature of *inflamed (tumor, CD8)* to be connected to the immune system whereas downregulated genes were shown to have a role DNA in mismatch repair. This supports the hypothesis that errors in DNA replication are the main driver for the immune answer in CRC.

Interestingly, these gene signatures can be seen as a representation of TIPs on the genomic level and allow the determination of TIPs also in data sources for which no IHC imaging data is available. We have demonstrated this approach in a CRC dataset from TCGA and showed that the predicted TIPs were associated with MSI-high tumors and the laterality of the tumor. The definition of TIPs on the genomic level, enable their use in a much broader range of data sources, e.g., in public databases or phase 3 clinical trials for which typically no IHC stainings are available.

In conclusion, we have demonstrated how tumor infiltration phenotypes can be quantified in CRC samples using a computational method that accounts for the high variability of spatial immune infiltration across the sample. The computational tumor infiltration phenotypes do not only have the potential to serve as biomarkers in immunotherapy but also enable a more detailed exploration of the immune response in CRC. Although immunotherapy has been successful in CRC in the last years, response rates are variable. In order to find the right treatment for every patient, a detailed characterization of individual tumors is necessary. Despite the established classifications for CRC, e.g., by consensus molecular subtypes or the MSI status, tumor infiltration phenotypes offer the potential for an even deeper characterization of CRC patients.

In the future, it would be of great interest to include more cell types into the analysis e.g., myeloid cells or fibroblasts to capture additional factors in the tumor microenvironment. Moreover, it remains to be shown whether TIPs can serve as prognostic or predictive biomarkers by testing their association to endpoints such as overall survival or treatment response.

## Data Availability Statement

The datasets presented in this study can be found in online repositories. The names of the repository/repositories and accession number(s) can be found in the article/Supplementary Material.

## Ethics Statement

Samples used in this study were provided by Avaden Biosciences, Seattle WA 98104, USA and Individumed GmbH, 20251 Hamburg, Germany. Written agreements for all relevant samples were signed, guaranteeing that appropriate ethics approvals were obtained.

## Author Contributions

FS and KK initiated the project. HF, FS, KK, AT, and NZ conceived the idea of computational tumor infiltration phenotypes. HF implemented the analysis. FS and AT provided statistical guidance. NZ and KK provided biological interpretation. HF and FS wrote the manuscript. HF, FS, KK, and AT revised the manuscript. All authors read and approved the submitted version.

## Conflict of Interest

HF, NZ, KK, and FS are employees of F. Hoffmann-La Roche Ltd. The remaining author declares that the research was conducted in the absence of any commercial or financial relationships that could be construed as a potential conflict of interest.
