# Supplementary material for: Computational Tumor Infiltration Phenotypes Enable the Spatial and Genomic Analysis of Immune Infiltration in Colorectal Cancer
Source: Front Oncol. 2021 Mar 15;11:552331. doi: 10.3389/fonc.2021.552331 (PMC8006941; doi:10.3389/fonc.2021.552331)
Supplement: Supplementary file 3 [file Data_Sheet_1.PDF]

Supplementary Material

A

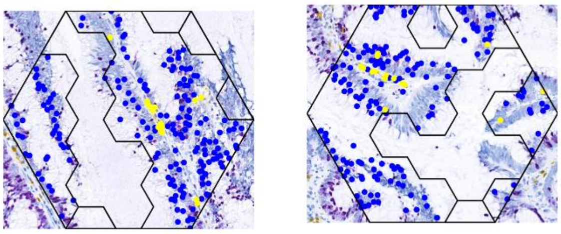

B

85% Accuracy

	Reference	
**Prediction**	excluded	inflamed
Excluded	48	7
Inflamed	11	52

Figure S1 (A) Preprocessing of image data. Empty regions in a tile are excluded from further analysis. (B) Accuracy of fused lasso model. The ground truth data is based on a KI67/CD8 duplex staining.

Table S 1 Multivariate model for the prediction of the microsatellite instability status (logistic regression).

	Dependent variable:
	IS_MSI
\`inflamed(tumor,CD8)\`	1.011^{**}
	(0.407)
\`Tumor Stage > IV\`	1.992^{**}
	(0.894)
\`CD8 Density\`	3.606^{*}
	(1.863)
\`CD4 Density\`	-1.228^{*}
	(0.745)
Age	-0.003
	(0.033)
Constant	-2.315
	(2.321)
Observations	80
Log Likelihood	-20.195
Akaike Inf. Crit.	52.390
Note:	*p<0.1; **p<0.05; ***p<0.01

Table S 2 Multivariate model for the prediction of tumor mutational burden.

	Dependent variable:
	TMB_score
`inflamed(tumor,CD8)`	6.894***
	(2.433)
`Tumor Stage > IV`	14.472***
	(4.936)
`CD8 Density`	3.882*
	(2.103)
`CD4 Density`	1.553
	(2.042)
Age	0.383**
	(0.161)
Constant	-14.364
	(11.191)
Observations	67
R^2	0.448
Adjusted R^2	0.403
Residual Std. Error	16.077 (df = 61)
F Statistic	9.920*** (df = 5; 61)
Note:	*p<0.1; **p<0.05; ***p<0.01

Table S 3 Multivariate model for the prediction of the Cytotoxic immune cell signature

	Dependent variable:
	` CYTOTOXIC CELLS`
`inflamed(tumor,CD8)`	1.399***
	(0.265)
`Tumor Stage > IV`	-0.181
	(0.542)
`CD8 Density`	0.406*
	(0.236)
`CD4 Density`	0.393*
	(0.225)
Age	0.012
	(0.017)
Constant	-0.918
	(1.170)
Observations	66
R^2	0.480
Adjusted R^2	0.437
Residual Std. Error	1.753 (df = 60)
F Statistic	11.099*** (df = 5; 60)
Note:	*p<0.1; **p<0.05; ***p<0.01

.

Table S 4 Multivariate model for the prediction of the TH1 immune cell signature.

	Dependent variable:
	` TH1`
`inflamed(tumor,CD8)`	2.644***
	(0.442)
`Tumor Stage > IV`	0.930
	(0.906)
`CD8 Density`	0.793**
	(0.395)
`CD4 Density`	0.902**
	(0.375)
Age	0.007
	(0.028)
Constant	-1.387
	(1.957)
Observations	66
R^2	0.595
Adjusted R^2	0.561
Residual Std. Error	2.931 (df = 60)
F Statistic	17.627*** (df = 5; 60)
Note:	*p<0.1; **p<0.05; ***p<0.01

Table S 5 Multivariate model for the prediction of the TH17 immune cell signature.

	Dependent variable:
	` TH17`
`inflamed(tumor,CD4)`	0.729***
	(0.197)
`Tumor Stage > IV`	0.641
	(0.422)
`CD8 Density`	-0.225
	(0.193)
`CD4 Density`	0.131
	(0.186)
Age	-0.005
	(0.014)
Constant	-0.093
	(0.976)
Observations	66
R^2	0.265
Adjusted R^2	0.203
Residual Std. Error	1.472 (df = 60)
F Statistic	4.316*** (df = 5; 60)
Note:	*p<0.1; **p<0.05; ***p<0.01

Table S 6 Genes set for mutation analysis

MLL3	APC	TP53	BRAF	PIK3CA
ARID1B	FAT3	SPEN	MLL2	ZNF217
BRCA2	LRP1B	GPR124	KRAS	GNAS
ASXL1	ATM	ARID1A	RNF43	

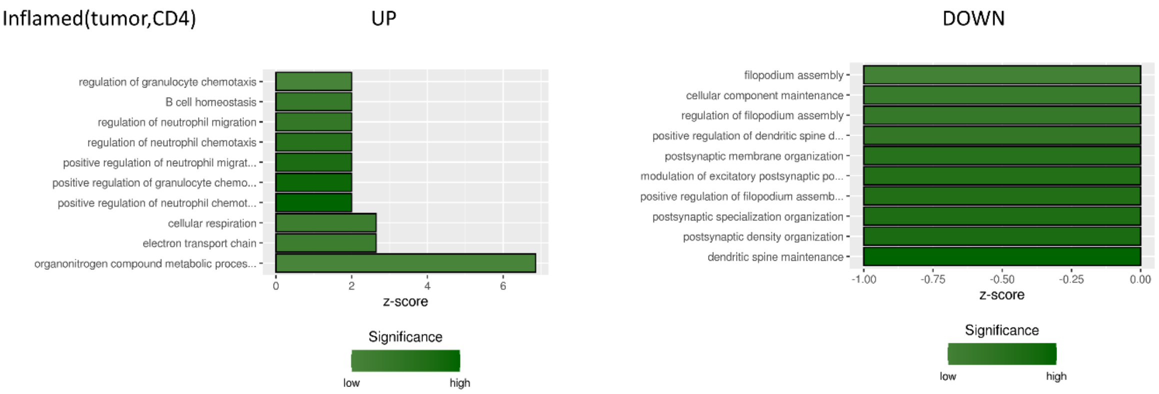

Figure S2 Gene set enrichment analysis fo ther gene signature of inflamed(tumor,CD4).

Table S7. Gene expression profile: inflamed(tumor,CD8)

Table S8. Gene expression profile: inflamed(tumor,CD4)

Supplementary Data S2. Point pattern data, CD8

Supplementary Data S3. Point pattern data, CD4

Supplementary Data S4. Point pattern data, tumor_1

Supplementary Data S5. Point pattern data, tumor_2

Supplementary Data S6. Example Script
