# Supplementary material for: Computational Tumor Infiltration Phenotypes Enable the Spatial and Genomic Analysis of Immune Infiltration in Colorectal Cancer
Source: Front Oncol. 2021 Mar 15;11:552331. doi: 10.3389/fonc.2021.552331 (PMC8006941; doi:10.3389/fonc.2021.552331)

Calculate Tumor Infiltration Phenotypes

Henrik Failmezger

20 01 2020

Process point pattern

Calculate Tumor Infiltration Phenotypes (TIPs) from point patterns of tumor and immune cells.

Load the required libraries:

```
rm(list = ls())
library(tidyverse)
library(spatstat)
library(penalized)
library(pracma)
library(caret)
```

Define the input paths:

```
# Set the working directory to the folder Scripts
pathToFolder=getwd()
#
pathToInputPP_CD8=file.path(pathToFolder,"PointPattern_CD8")
pathToInputPP_CD4=file.path(pathToFolder,"PointPattern_CD4")
pathToInputPP_tumor=file.path(pathToFolder,"PointPattern_tumor")
#
pathToInputModel=pathToFolder
#
```

Load the fused regression model for classification:

```
list_model=readRDS(file.path(pathToInputModel,"fusedLasso_penalized.RDS"))
model_flr=list_model$model
normParam= list_model$normParam
```

Define the parameters for classification:

```
paramRlow=1
paramR=338
windowSize=50
M_FROM="t"
M_TO="CD8"
image_id=301258
```

Load the script:

```
source(file.path(pathToFolder,"calculate_TIP.R"))
```

Compute tumor infiltration phenotype:

```
TIP_image=compute_TIP(image_id,pathToInputPP_CD8,pathToInputPP_tumor,pathToInputPP_CD4,M_FROM,
M_TO,paramR,paramRlow,model_flr,normParam)
```

```
## Warning in quadratcount.ppp(img_io, tess = tesselationHoles): Tessellation does
## not contain all the points of X
```

Plot the TIP-map:

```
plot(TIP_image$shiftsRatios[[1]]$tesselationShifted,do.col=T,main="")
```

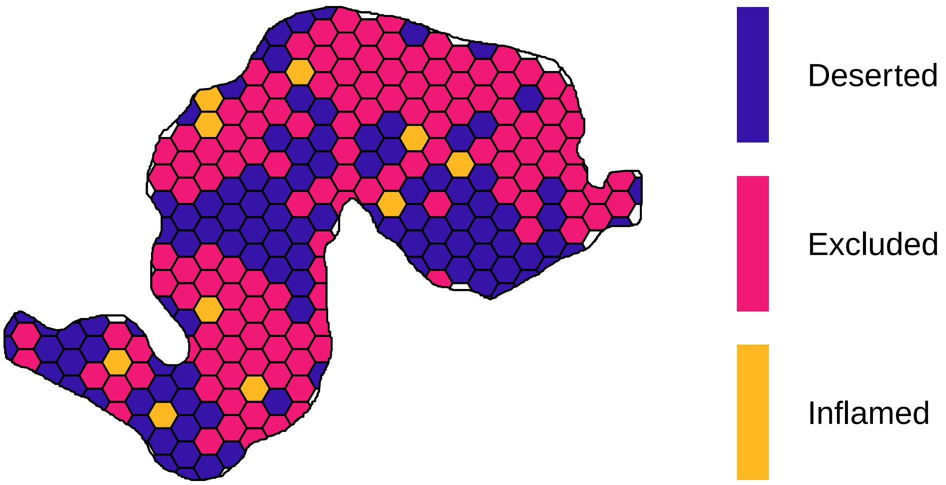

Supplement: Supplementary file 8 [file Data_Sheet_6.ZIP › Scripts/calculateTumorInfiltrationPhenotype.pdf]
